# Multimorbidity and the risk of malnutrition, frailty and sarcopenia in adults with cancer in the UK Biobank

**DOI:** 10.1002/jcsm.13523

**Published:** 2024-06-21

**Authors:** Nicole Kiss, Gavin Abbott, Robin M. Daly, Linda Denehy, Lara Edbrooke, Brenton J. Baguley, Steve F. Fraser, Abbas Khosravi, Carla M. Prado

**Affiliations:** ^1^ Institute for Physical Activity and Nutrition Deakin University Geelong Australia; ^2^ Department of Health Services Research Peter MacCallum Cancer Centre Parkville Australia; ^3^ Department of Physiotherapy University of Melbourne Parkville Australia; ^4^ Institute for Intelligent Systems Research and Innovation Deakin University Geelong Australia; ^5^ Department of Agricultural, Food and Nutritional Science University of Alberta Edmonton Canada

**Keywords:** cancer, frailty, malnutrition, multimorbidity, sarcopenia

## Abstract

**Background:**

Malnutrition, sarcopenia and frailty are distinct, albeit interrelated, conditions associated with adverse outcomes in adults with cancer, but whether they relate to multimorbidity, which affects up to 90% of people with cancer, is unknown. This study investigated the relationship between multimorbidity with malnutrition, sarcopenia and frailty in adults with cancer from the UK Biobank.

**Methods:**

This was a cross‐sectional study including 4122 adults with cancer (mean [SD] age 59.8 [7.1] years, 50.7% female). Malnutrition was determined using the Global Leadership Initiative on Malnutrition criteria. Probable sarcopenia and sarcopenia were defined using the European Working Group on Sarcopenia in Older People 2 criteria. (Pre‐)frailty was determined using the Fried frailty criteria. Multimorbidity was defined as ≥2 long‐term conditions with and without the cancer diagnosis included. Logistic regression models were fitted to estimate the odds ratios (ORs) of malnutrition, sarcopenia and frailty according to the presence of multimorbidity.

**Results:**

Genitourinary (28.9%) and breast (26.1%) cancers were the most common cancer diagnoses. The prevalence of malnutrition, (probable‐)sarcopenia and (pre‐)frailty was 11.1%, 6.9% and 51.2%, respectively. Of the 11.1% of participants with malnutrition, the majority (9%) also had (pre‐)frailty, and 1.1% also had (probable‐)sarcopenia. Of the 51.2% of participants with (pre‐)frailty, 6.8% also had (probable‐)sarcopenia. No participants had (probable‐)sarcopenia alone, and 1.1% had malnutrition, (probable‐)sarcopenia plus (pre‐)frailty. In total, 33% and 65% of participants had multimorbidity, including and excluding the cancer diagnosis, respectively. The most common long‐term conditions, excluding the cancer diagnosis, were hypertension (32.5%), painful conditions such as osteoarthritis or sciatica (17.6%) and asthma (10.4%). Overall, 80% of malnourished, 74% of (probable‐)sarcopenia and 71.5% of (pre‐)frail participants had multimorbidity. Participants with multimorbidity, including the cancer diagnosis, had higher odds of malnutrition (OR 1.72 [95% confidence interval, CI, 1.31–2.30; *P* < 0.0005]) and (pre‐)frailty (OR 1.43 [95% CI 1.24–1.68; *P* < 0.0005]). The odds increased further in people with ≥2 long‐term conditions in addition to their cancer diagnosis (malnutrition, OR 2.41 [95% CI 1.85–3.14; *P* < 0.0005]; (pre‐)frailty, OR 2.03 [95% CI 1.73–2.38; *P* < 0.0005]). There was little evidence of an association of multimorbidity with sarcopenia.

**Conclusions:**

In adults with cancer, multimorbidity was associated with increased odds of having malnutrition and (pre‐)frailty but not (probable‐)sarcopenia. This highlights that multimorbidity should be considered a risk factor for these conditions and evaluated during nutrition and functional screening and assessment to support risk stratification within clinical practice.

## Introduction

Globally, the incidence of cancer is increasing and is predicted to reach almost 25 million by the year 2030.^1^ Weight loss and low muscle mass are commonly associated with cancer diagnosis or treatment, as well as the metabolic effect of the tumour and systemic inflammation affecting nutritional status, among other physiological functions.^2^ This can lead to the development of malnutrition, sarcopenia and frailty, conditions that are distinct, albeit interrelated, and independently associated with an array of adverse outcomes, including reduced survival.^3^, ^4^, ^5^

Malnutrition affects approximately one in three people with cancer,^5^ with a higher prevalence of up to 60–80% in advanced stage disease and some diagnoses such as lung, head and neck and gastrointestinal cancers.^5^ Cancer‐related malnutrition is commonly associated with serious adverse outcomes, including reduced quality of life, poor physical function and higher mortality.^2^, ^5^ Less is known about the prevalence and impact of sarcopenia in cancer, as defined by a combination of low muscle strength with low muscle mass and/or poor physical performance, with results varying depending on the sarcopenia definition adopted.^6^, ^7^ To date, most research on patients with cancer has focused on the impact of low muscle mass alone.^3^, ^8^ Research has demonstrated that sarcopenia is independently associated with an increased risk of post‐operative complications and mortality in people with colorectal and gastric cancer.^9^, ^10^ Furthermore, we recently found that both malnutrition and sarcopenia were associated with a 2.5‐ and 2.9‐times higher hazard for all‐cause mortality in UK Biobank participants with cancer.^11^

There is a growing interest in the impact of frailty on outcomes during and following cancer treatment.^12^ Frailty is reported to affect between 16% and 42% of older people with cancer, depending on the cancer diagnosis and criteria used for the frailty diagnosis.^4^, ^12^, ^13^ Similar to malnutrition and sarcopenia, frailty is also associated with significant adverse consequences. For instance, in studies evaluating frailty in a variety of cancer types, being frail was associated with a 1.4‐ to 2.6‐times higher hazard of mortality.^4^, ^14^ The odds of experiencing treatment complications, poorer quality of life and a longer length of hospital stay were also significantly higher in frail patients compared with non‐frail patients with cancer.^12^, ^13^, ^15^

Previous studies conducted in the full UK Biobank cohort of approximately 500 000 participants have reported that the risk of (probable‐)sarcopenia and (pre‐)frailty is higher in people with multimorbidity.^16^, ^17^ Multimorbidity, the co‐occurrence of multiple long‐term health conditions, is reported to affect up to 91% of people with cancer, with 23% having five or more long‐term conditions.^18^ However, whether multimorbidity increases the risk of people with cancer being malnourished, sarcopenic or frail is yet to be investigated. Developing an understanding of the association between multimorbidity and the likelihood of being malnourished, sarcopenic or frail has important implications for risk stratification and the allocation of allied health services, given the impact of these conditions on adverse clinical outcomes. Therefore, this study aimed to investigate the relationship between multimorbidity and the risk of malnutrition, sarcopenia and frailty in UK Biobank participants with cancer.

## Methods

### Study population

The UK Biobank is a large epidemiological study involving over 500 000 adults aged 37–73 years from the United Kingdom.^19^ Recruited participants completed a baseline assessment between 2006 and 2010 at 22 centres across England, Wales and Scotland.^19^ To be eligible for our study, participants needed to be within 2 years of their cancer diagnosis at the time of the baseline assessment to account for the potential for malnutrition, sarcopenia and/or frailty to persist beyond treatment. Participants with non‐melanoma skin cancer were excluded. The criteria and definitions used for malnutrition, sarcopenia and frailty in this study were adapted (*Table* *1*) from data available to UK Biobank participants as previously described.^17^, ^22^ This paper is reported according to the criteria in the Strengthening the Reporting of Observational studies in Epidemiology (STROBE) guidelines.^23^

**Table 1:** Criteria used to determine malnutrition, sarcopenia and frailty adapted for the UK Biobank

Component	Original criteria	UK Biobank
Malnutrition		
Weight loss	>5% in the past 6 months or >10% beyond 6 months	Self‐reported: ‘Compared with one year ago, has your weight changed?’ Response: Yes, lost weight = 1 or other = 0 (no/yes, gained weight)
Low body mass index	*Mild to moderate*: <20 kg/m^2^ if <70 years or <22 kg/m^2^ if ≥70 years *Severe*: <18.5 kg/m^2^ if <70 years or <20 kg/m^2^ if ≥70 years	Per original criteria
Reduced muscle mass	*Mild to moderate* deficit by validated assessment methods	*Mild to moderate*: ALST/BMI of <0.64 (females) and <0.94 (males)
	*Severe* deficit by validated assessment methods	*Severe*: ALST/BMI of <0.55 (females) and <0.84 (males)
Inflammation	Acute disease/injury or chronic disease related	CRP > 5 mg/L
Reduced food intake or assimilation	≤50% energy intake for >1 week or any reduction for >2 weeks or any condition that adversely impacts food assimilation or absorption	Not available
Sarcopenia		
Low muscle strength	Handgrip strength	Per original criteria
	<27 kg (males)	
	<16 kg (females)	
Low muscle mass	ALST/ht^2^	ALST/BMI
	<7.0 kg/m^2^ (males)	<0.55 (females)
	<5.5 kg/m^2^ (females)	<0.84 (males)
Low physical performance	Gait speed	Self‐reported: ‘How would you describe your usual walking pace?’
	<0.8 m/s	Response: Slow = 1, other = 0 (average, brisk, none of the above)
Frailty		
Weight loss	Self‐reported: ‘In the last year have you lost ≥4.5 kg unintentionally?’	Self‐reported: ‘Compared with one year ago, has your weight changed?’ Response: Yes, lost weight = 1 or other = 0 (no, gained weight)
Exhaustion	Self‐reported: ‘How often in the past week (a) did you feel that everything was an effort, or (b) could you not get going?’	Self‐reported: ‘Over the past two weeks, how often have you felt tired or had little energy?’ Response: More than half the days or nearly every day = 1, other = 0 (not at all, several days)
Low physical activity	Self‐reported: Kcal of activity per week estimated using the Minnesota Leisure Time Activity Questionnaire. Lowest 20% identified as meeting frail criteria.	Self‐reported: Using the International Physical Activity Questionnaire Response: None or light activity with a frequency of once per week or less = 1, medium or heavy activity or light activity more than once per week = 0
Slow walking speed	Measured time to walk 4.5 m	Self‐reported: ‘How would you describe your usual walking pace?’ Response: Slow = 1, other = 0 (brisk, steady/average, none of the above)
Low grip strength	Handgrip strength (adjusted for sex and BMI) using cut‐offs from Fried et al.^20^	Per original criteria

*Note*: Malnutrition was assessed using the Global Leadership Initiative on Malnutrition (GLIM) criteria^21^; sarcopenia was assessed using the European Working Group on Sarcopenia in Older People 2 (EWGSOP2) definition^6^; and frailty was assessed using the Fried frailty criteria,^20^ adapted by Hanlon et al. for the UK Biobank.^17^ Abbreviations: ALST, appendicular lean soft tissue; BMI, body mass index; CRP, C‐reactive protein.

### Demographics and cancer diagnosis

Participants reported demographics (age and sex), ethnicity (White and other), smoking status (never, previous and current) and frequency of alcohol intake (daily or almost daily, one to four times per week, one to three times per month, never or on special occasions only) at the baseline assessment. The degree of economic deprivation was determined using Townsend deprivation scores, based on participant postcodes reported in the preceding national census.^24^ A higher degree of deprivation is represented by an increasing Townsend score.

Data linkage from national cancer registries was used to determine the date of cancer diagnosis and type of cancer according to the International Classification of Diseases 10th revision (ICD‐10). Cancers were grouped into 11 types: bone and soft tissue, breast, central and peripheral nervous system, endocrine and thyroid, gastrointestinal, genitourinary, haematological, head and neck, lung and other thoracic, melanoma and unknown primary. The time since cancer diagnosis was determined by the difference (years) between the date of cancer diagnosis and the date of baseline assessment.

### Assessment of malnutrition

The Global Leadership Initiative on Malnutrition (GLIM) criteria were applied to categorize malnutrition.^21^ The GLIM criteria require at least one of the three phenotypic criteria and one of the two aetiologic criteria to be met. The three phenotypic criteria include unintentional weight loss, low body mass index (BMI) and low muscle mass. The two aetiologic criteria include reduced food intake or assimilation and inflammation. The data used from the UK Biobank for the phenotypic and aetiologic criteria are described in *Table* *1*. Data to determine low muscle mass are described below and in accordance with published criteria.^25^, ^26^ Inflammation was used to evaluate the aetiologic criteria. Food intake data were not available for this sample, and therefore, only the inflammation aetiologic criterion was used.

### Assessment of sarcopenia

The European Working Group on Sarcopenia in Older People 2 (EWGSOP2) definition was applied to individuals of all ages to categorize secondary probable sarcopenia or sarcopenia related to the presence of cancer.^6^

#### Low muscle mass

Fat‐free mass (FFM) derived from bioelectrical impedance analysis (Tanita BC418MA, Tokyo, Japan) was used to estimate appendicular lean soft tissue (ALST) using an equation developed by Dodd et al., which used data from UK Biobank participants who, at a later assessment, completed a dual X‐ray absorptiometry (DXA) to assess ALST. The equation used is as follows: ALST (kg) = (0.958 × [Appendicular FFM (kg)]) − (0.166 × S) − 0.308, with S taking a value of 0 if female and 1 if male.^16^

Cut‐points specific to the UK Biobank were derived by our group using participants aged 45 years or less as the reference as previously described.^11^ Cut‐points of one standard deviation below the sex‐specific mean were used for mild to moderate low ALST and two standard deviations below the mean for severe low ALST, as required for the GLIM criteria. In a previous paper, we compared low ALST when ALST was adjusted for BMI (ALST/BMI) or height (ALST/ht^2^) and determined that both methods were associated with increased mortality; however, ALST/BMI identified a higher prevalence of cases, and therefore, ALST/BMI was used in this analysis (*Table* *1*).^27^ Regardless, the results were similar in this paper with either method. The term muscle mass is used throughout this paper when referring to ALST for simplicity.

#### Handgrip strength

A trained research nurse determined grip strength using a Jamar J00105 hydraulic hand dynamometer. Participants were seated in an upright position with 90° elbow flexion, with the forearm placed on armrests in the mid‐prone position. A single measurement was taken for each arm, and the maximum value of the two measurements was used to determine peak grip strength (kilograms).^6^

#### Physical performance

An objective measure of physical performance is not available within the UK Biobank; therefore, self‐reported walking pace was used as described in previous studies of sarcopenia in UK Biobank participants (*Table* *1*).^16^, ^17^

#### Diagnosis of probable sarcopenia and sarcopenia

As shown in *Table* *1*, participants were considered to have probable sarcopenia if handgrip strength was low, sarcopenia if they also had low muscle mass and severe sarcopenia if they also had poor physical performance. The term (probable‐)sarcopenia has been used throughout this paper when referring to probable sarcopenia and sarcopenia combined.

### Assessment of frailty

The Fried frailty criteria, comprising weight loss, exhaustion, low physical activity, slow walking speed and low grip strength, were used to categorize pre‐frailty and frailty.^20^ These measures were adapted for the UK Biobank by Hanlon et al.^17^ and used in this study as described in *Table* *1*. Although originally designed for older adults (≥65 years), frailty is known to affect younger adults and is indicative of phenotypic and biological age as opposed to chronological age.^17^ The cut‐points for low handgrip strength defined by Fried et al. were applied.^20^ These differ from those previously described for the assessment of sarcopenia as they were developed specifically for frailty and are adjusted for BMI in addition to sex (*Table* *S1*). Pre‐frailty is considered present if one or two criteria are met, and frailty is considered present if three or more criteria are met. The term (pre‐)frailty has been used throughout this paper to refer to pre‐frailty and frailty combined.

### Multimorbidity

Participants reported all non‐cancer health conditions at the baseline assessment. These illnesses were confirmed at a follow‐up interview by a research nurse and classified against a hierarchical tree of more than 450 conditions based on the ICD‐10 classification. Of these, long‐term conditions were defined from a list of 43 conditions described in a large epidemiological study in Scotland^28^ and previously amended for the UK Biobank (*Table* *S2*).^17^, ^29^ The number of long‐term conditions was classified as none, one, two, three or four or more, with multimorbidity defined as two or more long‐term conditions.^28^ Multimorbidity was examined with and without cancer, which was included as one of the long‐term conditions.

### Statistical analysis

Clinical and demographic characteristics were summarized for all participants using the mean (standard deviation) or median (interquartile range) for continuous variables and the count (percentage) for categorical variables. The prevalence (count [percentage]) of malnutrition, (probable‐)sarcopenia and (pre‐)frailty were summarized for all cancer diagnoses overall and by the number of long‐term conditions (0, 1, 2, 3 and ≥4). Binary logistic regression models (odds ratios and 95% confidence intervals) were used to explore the association between multimorbidity (1 or ≥2 long‐term conditions in addition to the cancer diagnosis) and binary outcomes such as malnutrition, (probable‐)sarcopenia and (pre‐)frailty. The absence of long‐term conditions was used as the reference group for the exposure variable. Separate models were fitted for malnutrition, (probable‐)sarcopenia and (pre‐)frailty and adjusted for age, sex, time since cancer diagnosis, deprivation score, smoking status and alcohol use. A sensitivity analysis was conducted in participants with a cancer diagnosis associated with a higher likelihood of metabolic alterations or cachexia, that is, lung and gastrointestinal cancers,^30^ in recognition of the potential impact on the outcomes. All analyses were conducted using Stata/MP 15.1 (StataCorp, TX, USA). The strength of the evidence against the null hypothesis (i.e., no association) was interpreted as follows: *P* < 0.001 very strong evidence, *P* < 0.01 strong evidence, *P* < 0.05 moderate evidence, *P* < 0.1 weak evidence and *P* > 0.1 insufficient evidence.^31^

## Results

**Figure 1:**
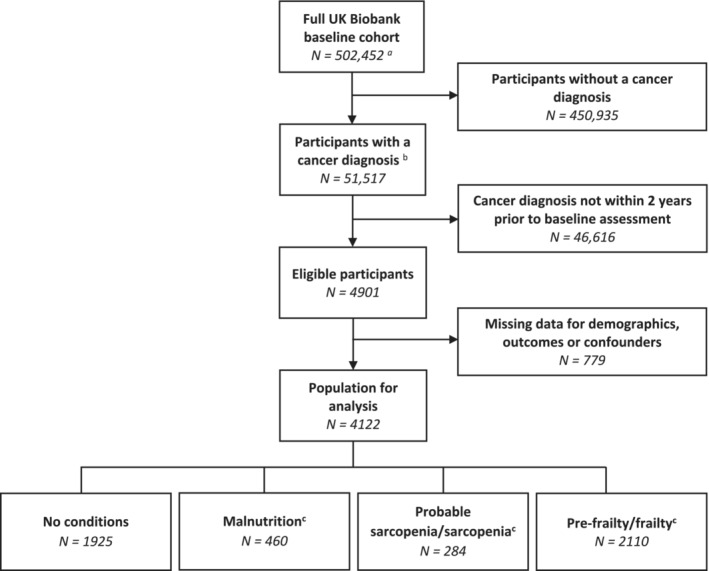
Study flow chart. ^a^Excludes participants who have requested their data be removed from the UK Biobank. ^b^Excludes non‐melanoma skin cancer and benign tumours. ^c^Participants may have had more than one of these conditions, and therefore, these numbers add up to more than the total number of participants.

After excluding participants without a diagnosis of cancer within 2 years prior to the baseline assessment and those with missing data for malnutrition, (probable‐)sarcopenia, (pre‐)frailty or covariates (*n* = 779), 4122 (0.8%) of the original 502 493 UK Biobank participants were included in this analysis (*Figure* *1*).

**Figure 2:**
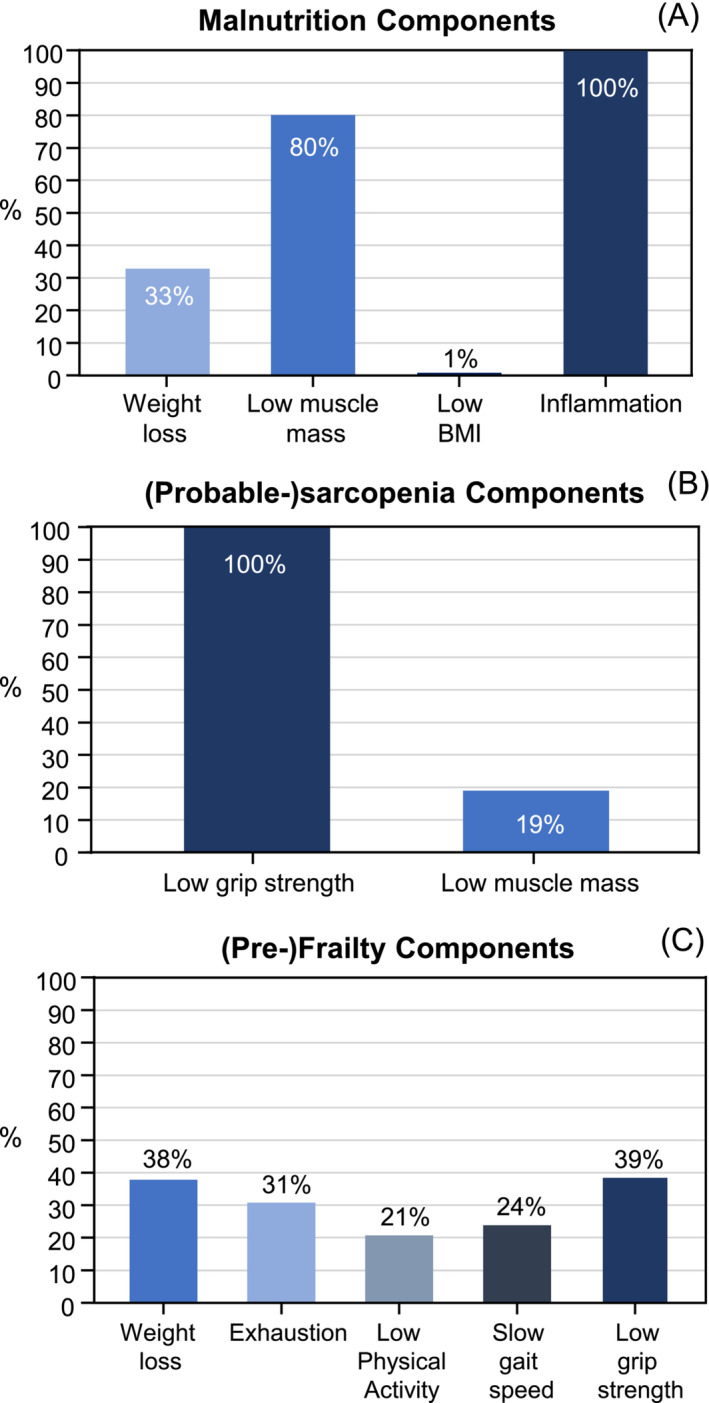
Proportion of participants with cancer meeting the underlying components of malnutrition (A), (pre‐)frailty (B) and (probable‐)sarcopenia (C) in those classified as having the condition. BMI, body mass index.

In the full cohort of 4122 participants, genitourinary (28.9%) and breast (26.1%) cancers were the most common diagnoses (*Table* *2*). The estimated prevalence of malnutrition was 11.1% (*n* = 460). All participants with malnutrition had inflammation because this was required to meet the GLIM aetiologic criteria, while low muscle mass was the most common of the three phenotypic criteria (*Figure* *2*). A total of 51.2% (*n* = 2110/4122) were (pre‐)frail, with 12% (*n* = 250/2110) having frailty. Low grip strength and weight loss were the most common of the five frailty criteria (*Figure* *2*). In total, (probable‐)sarcopenia had an estimated prevalence of 6.9% (*n* = 284/4122). Of the 284 participants with (probable‐)sarcopenia, 19% (*n* = 54/284) had sarcopenia (*Figure* *2*). The majority of participants who were malnourished, sarcopenic or (pre‐)frail were <65 years old (*Table* *2*). However, the prevalence of each of these conditions was higher in those 65 years of age or older compared with those under 65 years of age (malnutrition: 156/1280 = 12.2% vs. 304/2842 = 10.6%; (probable‐)sarcopenia: 122/1280 = 9.5% vs. 162/2842 = 5.7%; (pre‐)frailty: 680/1280 = 53.1% vs. 1430/2842 = 50.3%). Overall, 33% (*n* = 1349/4122) of participants were considered to have multimorbidity (two or more long‐term conditions) in addition to cancer. Including the cancer diagnosis as one of the long‐term conditions, 65% (*n* = 2678/4122) of participants had multimorbidity. The most common long‐term conditions were hypertension (*n* = 1339/4122), ‘painful conditions’ (*n* = 727/4122), which encompassed conditions such as back pain, sciatica and osteoarthritis (*Table* *S2*), and asthma (*n* = 427/4122). In comparison to the full cohort, participants with malnutrition were heavier, while participants with sarcopenia were older (*Table* *2*). Genitourinary and breast cancers remained the most common cancer diagnoses among participants with malnutrition, (probable‐)sarcopenia and (pre‐)frailty.

**Table 2:** Participant characteristics for the full cohort and participants with no conditions, malnutrition, (probable‐)sarcopenia and (pre‐frailty) (*N* = 4122)

Characteristic	Full cohort	No conditions	Malnutrition	(Probable‐)sarcopenia	(Pre‐)frailty
	*N* = 4122	*n* = 1925	*n* = 460	*n* = 284	*n* = 2110
	Mean ± SD or *n* (%)	Mean ± SD or *n* (%)	Mean ± SD or *n* (%)	Mean ± SD or *n* (%)	Mean ± SD or *n* (%)
Age (years)	59.8 ± 7.1	59.6 (7.2)	60.8 ± 6.5	62.0 ± 6.4	60.0 ± 7.1
Age category (years)					
<65	2842 (68.9)	1355 (70.4)	304 (66.0)	162 (57.0)	1430 (67.8)
≥65	1280 (31.1)	570 (29.6)	156 (34.0)	122 (43.0)	680 (32.2)
Sex					
Male	2031 (49.3)	996 (51.7)	221 (48.0)	139 (48.9)	984 (46.6)
Female	2091 (50.7)	929 (48.3)	239 (52.0)	145 (51.1)	1126 (53.3)
Cancer diagnosis					
Genitourinary	1192 (28.9)	635 (33.0)	101 (21.9)	93 (32.7)	532 (25.2)
Breast	1075 (26.1)	503 (26.1)	92 (20.0)	78 (27.5)	556 (26.3)
Gastrointestinal	607 (14.7)	241 (12.5)	90 (19.6)	46 (16.2)	350 (16.6)
Haematological	311 (7.5)	133 (6.9)	38 (8.3)	20 (7.0)	173 (8.2)
Gynaecological	298 (7.2)	122 (6.3)	46 (10.0)	18 (6.3)	170 (8.0)
Melanoma	288 (7.0)	167 (8.7)	22 (4.7)	10 (3.5)	116 (5.5)
Head and neck	118 (2.9)	34 (1.8)	16 (3.5)	8 (2.8)	84 (4.0)
Lung and other thoracic	82 (2.0)	23 (1.2)	33 (7.2)	6 (2.1)	53 (2.5)
Endocrine and thyroid	56 (1.4)	29 (1.5)	5 (1.1)	0 (0)	27 (1.3)
Central and peripheral nervous system	37 (0.9)	18 (0.9)	5 (1.1)	3 (1.1)	18 (0.8)
Bone and soft tissue	31 (0.7)	16 (0.8)	5 (1.1)	1 (0.4)	12 (0.6)
Unknown primary	27 (0.7)	6 (0.3)	7 (1.5)	1 (0.4)	19 (1.0)
Ethnic background					
White	3984 (96.6)	1876 (97.4)	448 (97.4)	266 (93.7)	2023 (95.8)
Non‐White	122 (3.0)	39 (2.0)	12 (2.6)	16 (5.6)	81 (3.8)
Prefer not to say	16 (0.4)	10 (0.6)	0 (0)	2 (0.7)	6 (0.4)
Weight (kg)	78.6 ± 15.7	77.3 ± 14.2	83.9 ± 19.2	77.0 ± 15.5	79.6 ± 16.8
BMI category					
<18.5	18 (0.4)	3 (0.2)	7 (1.5)	4 (1.5)	15 (0.7)
18.5–24.9	1298 (31.5)	722 (37.5)	78 (17.0)	77 (27.1)	564 (26.7)
25.0–29.9	1776 (43.1)	873 (45.3)	148 (32.2)	122 (42.9)	869 (41.2)
≥30	1030 (25.0)	327 (17.0)	227 (49.3)	81 (28.5)	662 (31.4)
Number of long‐term conditions					
0	1444 (35.0)	822 (42.7)	92 (20.0)	74 (26.0)	602 (28.5)
1	1329 (32.2)	618 (32.1)	147 (32.0)	92 (32.4)	680 (32.2)
2	764 (18.5)	320 (16.6)	115 (25.0)	55 (19.4)	419 (19.8)
3	353 (8.7)	108 (5.6)	66 (14.3)	35 (12.3)	237 (11.2)
≥4	232 (5.6)	57 (3.0)	40 (8.7)	28 (9.9)	172 (8.3)
Multimorbidity (≥2 long‐term conditions)					
Including cancer diagnosis	2678 (75.0)	1103 (57.3)	368 (80.0)	210 (74.0)	1508 (71.5)
Excluding cancer diagnosis	1349 (32.8)	485 (25.2)	221 (48.0)	118 (41.6)	828 (39.3)

Abbreviation: BMI, body mass index.

### Prevalence and overlap between malnutrition, sarcopenia and frailty

**Figure 3:**
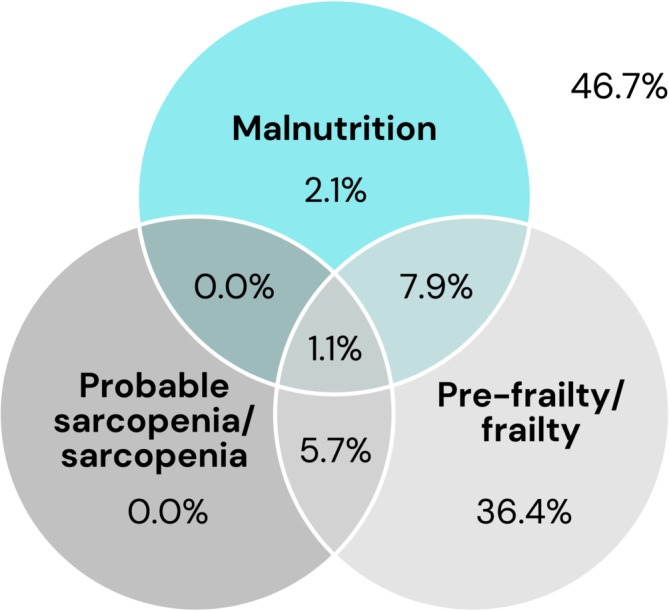
Venn diagram representing the overlap between the prevalence of malnutrition, probable sarcopenia/sarcopenia and pre‐frailty/frailty. Malnutrition only = 2.1%, malnutrition + (probable‐)sarcopenia = 0.0%, malnutrition + (pre‐)frailty = 7.9%, (probable‐)sarcopenia only = 0.0%, (probable‐)sarcopenia + (pre‐)frailty = 5.7% and malnutrition + (probable‐)sarcopenia + (pre‐)frailty = 1.1%. The number outside the figure represents the proportion of participants (46.7%) with none of these conditions.

Of the 11.1% of participants with malnutrition, the majority (9%) also had (pre‐)frailty, and 1.1% also had (probable‐)sarcopenia (*Figure* *3*). Of the 51.2% of participants with (pre‐)frailty, 6.8% also had (probable‐)sarcopenia. No participants had (probable‐)sarcopenia in isolation, while 1.1% of participants had all three conditions.

### Prevalence of malnutrition, sarcopenia and frailty with increased multimorbidity

**Figure 4:**
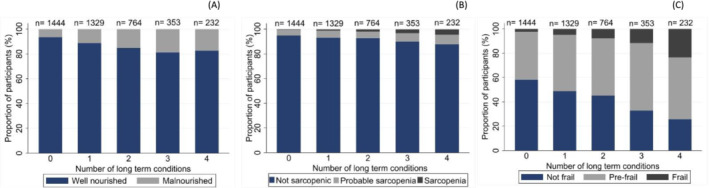
Prevalence of (A) malnutrition, (B) sarcopenia and (C) frailty by number of long‐term conditions (*N* = 4122).

The prevalence of malnutrition, (pre‐)frailty and (probable‐)sarcopenia all increased with increasing multimorbidity, with the increase most noticeable in participants with (pre‐)frailty (*Figure* *4*). Overall, 80% (*n* = 368/460) of malnourished participants, 74% (*n* = 210/284) of participants with (probable‐)sarcopenia and 71.5% (*n* = 1508/2110) of those who were (pre‐)frail had multimorbidity when their cancer diagnosis was included as one of the long‐term conditions (*Table* *2*). When the cancer diagnosis was excluded, that is, at least two long‐term conditions were present in addition to the cancer diagnosis, 48% (*n* = 221/460) of malnourished participants, 41.6% (*n* = 118/284) of participants with (probable‐)sarcopenia and 39.3% (*n* = 828/2110) of those who were (pre‐)frail had multimorbidity. Among participants with four or more long‐term conditions, 74% (*n* = 172/232) were (pre‐)frail.

### Association between malnutrition, sarcopenia and frailty with multimorbidity

There was very strong evidence of associations between increasing multimorbidity (long‐term conditions in addition to cancer) and higher odds of malnutrition and (pre‐)frailty (*Table* *3*). Participants with one long‐term condition in addition to cancer had an estimated 1.72‐times higher odds of malnutrition and 1.43‐times higher odds of (pre‐)frailty. The odds of these outcomes being present further increased in people who had ≥2 long‐term conditions in addition to their cancer diagnosis, with 2.41‐times higher odds of malnutrition and 2.03‐times higher odds of pre‐frailty/frailty. There was insufficient to weak evidence of an association between (probable‐)sarcopenia and multimorbidity, with similar results when probable sarcopenia and sarcopenia were separated (*Table* *S3*). These results were similar in participants with a cancer diagnosis with a high likelihood of cachexia (*Table* *S4*).

**Table 3:** Odds ratio (OR) of having malnutrition, (probable‐)sarcopenia or (pre‐)frailty by increasing multimorbidity in all adults with cancer (*N* = 4122)

Condition	One long‐term condition in addition to the cancer diagnosis^a^			Two or more long‐term conditions in addition to the cancer diagnosis^a^		
	OR	95% CI	*P*‐value	OR	95% CI	*P*‐value
Malnutrition	1.72	(1.31, 2.30)	<0.0005	2.41	(1.85, 3.14)	<0.0005
Pre‐frailty/frailty	1.43	(1.24, 1.68)	<0.0005	2.03	(1.73, 2.38)	<0.0005
Probable sarcopenia/sarcopenia	1.21	(0.88, 1.67)	0.239	1.34	(0.98, 1.83)	0.066

Abbreviation: CI, confidence interval.

^a^ The reference category was no long‐term conditions. All ORs were adjusted for age, sex, time since cancer diagnosis, smoking status, alcohol intake and deprivation score.

## Discussion

To our knowledge, this is the first study to investigate the association between multimorbidity and the risk of malnutrition, sarcopenia and frailty in people with cancer. In this cohort of 4122 adults with cancer from the UK Biobank, we found that over half (51.2%) had (pre‐)frailty, while the prevalence of malnutrition (11.1%) and (probable‐)sarcopenia (6.9%) were lower. Two thirds of participants had one or more conditions in addition to their cancer diagnosis (multimorbidity). The prevalence of malnutrition, (probable‐)sarcopenia and (pre‐)frailty all increased with increasing multimorbidity, while among participants with four or more long‐term conditions, three quarters were (pre‐)frail. Furthermore, multimorbidity increased the odds of being malnourished by 1.72–2.41 times and (pre‐)frail by 1.43–2.03 times, with higher odds as the degree of multimorbidity increased. However, there was limited evidence that multimorbidity was associated with higher odds of (probable‐)sarcopenia.

A systematic review of 20 observational studies in older adults with any stage of solid or haematological malignancy found frailty, assessed using the Fried criteria or geriatric assessment, to be associated with a 1.8‐times higher mortality hazard, a 2.6‐times increased hazard of chemotherapy intolerance and almost 5‐times higher odds of post‐operative complications.^12^ Frailty is a condition largely associated with ageing, and thus, current studies on frailty in people with cancer have focused on older adults (65 years of age and older).^32^ Our study has demonstrated a high prevalence of (pre‐)frailty (50.3%) in a younger cohort of UK Biobank participants with cancer, where almost 70% of the sample were aged <65 years. Similarly, a previous study using data from the full UK Biobank cohort of adults aged 37–73 years reported the prevalence of (pre‐)frailty to be 41%, which indicates that (pre‐)frailty can also affect middle‐aged people.^17^ Notably, our study found that the prevalence of (pre‐)frailty was higher at 51% among UK Biobank participants within 2 years of a cancer diagnosis, suggesting that it is more common among people with a recent cancer diagnosis. This is consistent with previous research where a prevalence of (pre‐)frailty of up to 50% has been reported at the time of a cancer diagnosis, albeit in older adults.^12^ While it has been suggested that the adapted frailty phenotype used in UK Biobank studies may differ clinically and biologically from the frailty phenotype present in older adults, defining (pre‐)frailty using the adapted phenotype has been associated with more than two‐times higher mortality hazard in UK Biobank participants of all ages.^17^ Furthermore, factors leading to the development of frailty may present at a younger age^17^; therefore, early identification of pre‐frailty as a precursor to frailty in people with cancer under 65 years old is important to guide appropriate care.

The high prevalence of (pre‐)frailty in comparison to malnutrition in this study may be explained by the need for only one of the frailty criteria to be met for a participant to be considered (pre‐)frail, as opposed to the requirement to meet at least one GLIM aetiologic plus one phenotypic criteria to be considered malnourished. This is also reflected in the finding that the majority of malnourished participants also had (pre‐)frailty; however, the majority of participants with (pre‐)frailty were not malnourished. Consequently, this highlights a vulnerable group of people with cancer and (pre‐)frailty who may benefit from nutrition intervention but will not be identified using current validated nutrition screening tools that focus on identifying malnutrition.^33^ In contrast, all participants with (probable‐)sarcopenia also had (pre‐)frailty. This was due to low grip strength being a common component of both diagnoses. Additionally, the cut‐points that denote low grip strength using the Fried frailty criteria are higher for both sexes than those used in the EWGSOP2 definition of sarcopenia and also increase at higher BMIs.^6^, ^20^ Low grip strength was the most common of the five frailty indicators among participants with (pre‐)frailty, followed by weight loss and exhaustion. This is similar to findings in the full UK Biobank cohort, where weight loss was the most common of the frailty indicators, followed by low grip strength and exhaustion.^17^

Participants with multimorbidity had significantly increased odds of malnutrition and (pre‐)frailty. These findings have important clinical implications for nutrition, functional screening and appropriate intervention. People with multimorbidity are likely to have complex nutritional and functional needs over and above the usual impact of a cancer diagnosis and treatment.^18^, ^28^ This is even more concerning when we consider that the odds of malnutrition and (pre‐)frailty being present are further increased if two or more long‐term conditions, in addition to the cancer diagnosis, are present, a situation that affected one third of participants in our study. A previous study in the full UK Biobank cohort found 1.13‐times higher odds of pre‐frailty and 1.53‐times higher odds of frailty in participants with a history of cancer, as opposed to within 2 years of a cancer diagnosis, also using the adapted Fried frailty criteria.^17^ The higher odds observed are likely due to including participants within 2 years of a cancer diagnosis, indicating that the recency of the cancer diagnosis is important for risk stratification. To our knowledge, our study is the first to demonstrate that multimorbidity, alongside a diagnosis of cancer, is associated with higher rates of malnutrition and (pre‐)frailty. Certain cancer diagnoses associated with high nutritional risk, such as head and neck or gastrointestinal cancers, or patients receiving high‐risk treatment types are often prioritized for nutritional and functional intervention by allied health professionals.^5^ Collectively, the findings from our study suggest that people with cancer who have multimorbidity have higher odds of malnutrition and (pre‐)frailty, and efforts to identify multimorbidity alongside current nutrition and functional screening should be considered. In health services where routine screening is not implemented, people with one or more long‐term conditions, in addition to their cancer diagnosis, should be assessed for malnutrition and frailty.

Interestingly, we found limited evidence of an association between multimorbidity and (probable‐)sarcopenia, despite all participants with (probable‐)sarcopenia also being (pre‐)frail and some also being malnourished. This may reflect the dominant components of malnutrition, (probable‐)sarcopenia and (pre‐)frailty diagnoses in this cohort, with only 19% of participants with (probable‐)sarcopenia having low muscle mass. In contrast, the vast majority of participants with malnutrition had low muscle mass and all had inflammation, both of which are known to be associated with the presence of chronic health conditions.^34^, ^35^ Among participants with (pre‐)frailty, low muscle mass was not part of the diagnostic criteria; however, the components that comprise the criteria have been selected to reflect musculoskeletal decline, chronic undernutrition and the impact of chronic illness on multiple physiologic systems.^20^ A previous study in the full UK Biobank cohort found 1.96‐times higher odds of probable sarcopenia, using the EWGSOP2 definition, in participants with multimorbidity.^16^ An alternative possibility is that the smaller sample size in our cohort of participants within 2 years of cancer diagnosis did not provide sufficient power to detect an association with multimorbidity, particularly as the prevalence of (probable‐)sarcopenia was the lowest out of the three conditions.

Multimorbidity is known to have a higher prevalence among people with obesity.^36^ In our cohort, 25% of participants had obesity, with the prevalence highest in participants with malnutrition (49.3%), followed by those with (pre‐)frailty (31.4%) and sarcopenia (28.5%). In this context, the ability to identify malnutrition, sarcopenia and frailty in this population is critical. BMI is known to be an unreliable indicator of malnutrition,^37^ while underlying muscle loss has been found to occur in people with cancer despite weight stability.^38^ The flexibility of the GLIM approach where one of three phenotypic criteria, low BMI, reduced muscle mass or unintentional weight loss, alongside one of two aetiologic criteria is more likely to support the identification of malnutrition in people with obesity; however, this requires investigation^21^, ^39^ and may be specifically due to the low reduced muscle mass.^25^ The components that comprise the Fried frailty criteria—weight loss, exhaustion, low physical activity and slow walking speed—are independent of BMI, while the cut‐points for low handgrip strength increase as BMI increases, suggesting its suitability for use in people with obesity.^20^ However, studies in older adults using the Fried criteria demonstrate conflicting results in regard to an association between obesity and frailty, with one study finding frailty to be associated with a 4.8‐times higher likelihood of a high waist circumference^40^ and another study finding obesity not associated with frailty, suggesting that further investigation is required.^41^ Similarly, low handgrip strength and low muscle mass, as part of the EWGSOP2 definition of probable sarcopenia and sarcopenia, are also independent of BMI.

The strengths of this study are the large sample size and broad age range of participants, which allowed us to investigate (pre‐)frailty in a younger cancer population than previous studies, and our analyses, which adjusted for multiple confounders. However, there are several limitations. UK Biobank participants are less representative of the general population, being predominantly White and less socio‐economically deprived.^42^ Furthermore, our findings may not be representative of the general cancer population because there is evidence of healthy volunteer selection bias.^42^ Consequently, people with cancer who are more unwell may not be represented, and therefore, prevalence estimates for malnutrition, (pre‐)frailty and (probable‐)sarcopenia may be underestimated in comparison to other cancer populations. Adapted outcomes for malnutrition and frailty were used, making the comparison with previous research challenging; however, these were based on previously validated adaptations where possible.^16^, ^17^ Retrospective application of the GLIM criteria meant available weight loss data did not specify if the weight loss was intentional or unintentional, and food intake could not be assessed for this sample. Low ALST, used within the malnutrition and sarcopenia diagnoses, was first estimated from bioelectrical impedance analysis and was secondly derived using a prediction equation that was developed in a non‐cancer population, both of which have the potential for measurement error and misclassification. Potentially important confounders, such as cancer stage and type of treatment, were not available and were therefore unable to be accounted for in the analyses.

In summary, we found a high prevalence of (pre‐)frailty (51.2%) and multimorbidity (33.0%) in UK Biobank participants within 2 years of a cancer diagnosis, but a lower prevalence of malnutrition (11.1%) and (probable‐)sarcopenia (6.9%). Our cohort of cancer participants with multimorbidity had 1.43‐ to 2.41‐times increased odds of having malnutrition and (pre‐)frailty, but there was limited evidence for increased odds of (probable‐)sarcopenia. However, this is in contrast to previous research in the full UK Biobank cohort, which has found multimorbidity to be associated with sarcopenia. In view of the increased risk of mortality associated with malnutrition and (pre‐)frailty, multimorbidity should be considered a risk factor for both conditions and evaluated and documented during routine nutritional and functional screening in people with cancer to support risk stratification and prioritization of people requiring nutrition and functional interventions.

## Funding

Dr Kiss and this study are supported by a Victorian Cancer Agency Nursing and Allied Health Clinical Research Fellowship (Grant No. CRFNAH18001).

## Conflict of interest statement

C.M.P. reports receiving honoraria and/or paid consultancy from Abbott Nutrition, Nutricia, Nestle Health Science, Fresenius Kabi, Pfizer and AMRA Medical. N.K., L.D., S.F.F., L.E., B.J.B., G.A., R.M.D. and A.K. have no conflicts of interest to report.

## Supporting information


**Table S1.** Cut‐points for low handgrip strength from Fried et al. [1].
**Table S2.** Long‐term conditions from UK Biobank.
**Table S3.** Odds ratio (OR) of having malnutrition, probable sarcopenia, sarcopenia, (pre‐)frailty by increasing multi‐morbidity in all adults with cancer (*N* = 4122).
**Table S4.** Odds ratio (OR) of having malnutrition, (probable‐)sarcopenia, (pre‐)frailty by increasing multi‐morbidity in adults with a high cachexia risk cancer (*N* = 689).
