# Supplementary material for: Multimorbidity and the risk of malnutrition, frailty and sarcopenia in adults with cancer in the UK Biobank
Source: J Cachexia Sarcopenia Muscle. 2024 Jun 21;15(5):1696–707. doi: 10.1002/jcsm.13523 (PMC11446695; doi:10.1002/jcsm.13523)
Supplement: Supplementary file 1 — Table S1. Cut‐points for low handgrip strength from Fried et al. [1]. Table S2. Long‐term conditions from UK Biobank. Table S3. Odds ratio (OR) of having malnutrition, probable sarcopenia, sarcopenia, (pre‐)frailty by increasing multi‐morbidity in all adults with cancer (N = 4122). Table S4. Odds ratio (OR) of having malnutrition, (probable‐)sarcopenia, (pre‐)frailty by increasing multi‐morbidity in adults with a high cachexia risk cancer (N = 689). [file JCSM-15-1696-s001.docx]

**Does multimorbidity increase the risk for malnutrition, frailty and sarcopenia in adults with cancer in the UK Biobank?**

**Kiss N et al.**

***Note: this supplementary material is intended for publication.***

**Supplementary Table 1: Cut-points for low handgrip strength from Fried et al. [1]**

| **Men** | **Cut-point** |
| --- | --- |
| BMI < 24 kg/m^2^ | < 29 |
| BMI 24.1 – 26 kg/m^2^ | < 30 |
| BMI 26.1 – 28 kg/m^2^ | < 30 |
| BMI > 28 kg/m^2^ | < 32 |
| **Women** |  |
| BMI < 23 kg/m^2^ | < 17 |
| BMI 23.1 – 26 kg/m^2^ | < 17.3 |
| BMI 26.1 – 29 kg/m^2^ | < 18 |
| BMI > 29 kg/m^2^ | < 21 |

1. Fried, L.P., Tangen C.M., Walston J, Newman A.B., Hirsch C, Gottdiener J et al., *Frailty in older adults: evidence for a phenotype.* J Gerontol A Biol Sci Med Sci, 2001. **56**(3): p. M146-56.

**Supplementary Table 2:** **Long-term conditions from UK Biobank**

| **Morbidity grouping** | **Conditions included (from UKB)** | **Code** |
| --- | --- | --- |
| **Painful conditions (1)** | Back pain  Joint pain  Headaches (not migraine)  Sciatica  Plantar fasciitis  Carpal tunnel syndrome  Fibromyalgia  Arthritis  Shingles  Disc problem  Prolapsed disc/slipped disc  Spine arthritis/spondylitis  Ankylosing spondylitis  Back problem  Osteoarthritis  Gout  Cervical spondylosis  Trigeminal neuralgia  Disc degeneration  Trapped nerve/compressed nerve | 1534  1537  1436  1476  1540  1541  1542  1538  1573  1532  1312  1311  1313  1294  1465  1466  1478  1523  1533  1257 |
| **Hypertension (2)** | Hypertension  Essential hypertension | 1065  1072 |
| **Depression (3)** | Depression  Postnatal depression | 1286  1531 |
| **Asthma (4)** | Asthma | 1111 |
| **Coronary Heart Disease (5)** | Heart attack/MI  Angina | 1075  1074 |
| **Treated dyspepsia (6)** | Gastro-oesophageal reflux (GORD)/gastric reflux  Oesophagitis /Barrett's oesophagus  Gastric stomach ulcers  Gastric erosions/gastritis  Duodenal ulcer  Dyspepsia/indigestion  Hiatus hernia  Helicobacter pylori | 1138  1139  1142  1143  1457  1510  1474  1442 |
| **Diabetes (7)** | Diabetic nephropathy  Diabetic neuropathy/ulcers  Diabetes  Type 1 diabetes  Type 2 diabetes  Diabetic eye disease | 1607  1468  1220  1222  1223  1276 |
| **Thyroid disorders (8)** | Thyroid problem (not cancer)  Hyperthyroidism/thyrotoxicosis  Hypothyroidism/myxoedema  Graves’ disease  Thyroid goitre  Thyroiditis | 1224  1225  1226  1522  1610  1428 |
| **Rheumatoid arthritis, other inflammatory polyarthropathies, systemic connective tissue disorders and systemic autoimmune disorders (9)** | Myositis/myopathy  Systemic Lupus Erythematosus  Connective tissue disorder  Sjogren’s syndrome/sicca syndrome  Dermatopolymyositis  Scleroderma/systemic sclerosis  Rheumatoid arthritis  Psoriatic arthropathy  Dermatomyositis  Polymyositis  Polymyalgia Rheumatica  Malabsorption/coeliac disease | 1322  1381  1373  1382  1383  1384  1464  1477  1383  1481  1377  1456 |
| **Chronic Obstructive Pulmonary Disease (COPD) (10)** | COPD/chronic obstructive airways disease  Emphysema/chronic bronchitis  Emphysema | 1112  1113  1472 |
| **Anxiety, other neurotic, stress related and somatoform disorders (11)** | Anxiety/panic attacks  Nervous breakdown  Post-traumatic stress disorder  Obsessive compulsive disorder  Stress  Insomnia  Psychological/psychiatric problem | 1287  1288  1469  1615  1614  1616  1243 |
| **Irritable bowel syndrome (12)** | Irritable bowel syndrome | 1154 |
| **Alcohol problems (13)** | Alcohol dependency  Alcoholic liver disease/alcoholic cirrhosis | 1408  1604 |
| **Other psychoactive substance abuse (14)** | Opioid dependency  Other substance abuse/dependency | 1409  1410 |
| **Treated constipation (15)** | Constipation | 1599 |
| **Stroke and Transient Ischaemic Attack (TIA) (16)** | Stroke  TIA  Subarachnoid haemorrhage  Brain haemorrhage  Ischaemic stroke | 1081  1082  1086  1491  1583 |
| **Chronic kidney disease (17)** | Polycystic kidney  Diabetic nephropathy  Renal/kidney failure  Renal failure requiring dialysis  Renal failure not requiring dialysis  Kidney nephropathy  Immunoglobulin A (IgA) nephropathy | 1427  1607  1192  1193  1194  1519  1520 |
| **Diverticular disease of intestine (18)** | Diverticular disease/diverticulitis | 1458 |
| **Atrial fibrillation (19)** | Atrial fibrillation | 1471 |
| **Peripheral vascular disease (20)** | Peripheral vascular disease  Leg claudication/intermittent claudication | 1067  1087 |
| **Heart failure (21)** | Cardiomyopathy  Hypertrophic cardiomyopathy  Heart failure/pulmonary oedema | 1079  1588  1076 |
| **Prostate disorders (22)** | Prostate problem (not cancer)  Enlarged prostate  Benign prostatic hypertrophy | 1207  1396  1516 |
| **Glaucoma (23)** | Glaucoma | 1277 |
| **Epilepsy (24)** | Epilepsy | 1264 |
| **Dementia (25)** | Dementia/Alzheimer/cognitive impairment | 1263 |
| **Schizophrenia (and related non-organic psychosis) and bipolar disorder (26)** | Schizophrenia  Mania/bipolar disorder/manic depression | 1289  1291 |
| **Psoriasis or eczema (27)** | Eczema/dermatitis  Psoriasis | 1452  1453 |
| **Inflammatory bowel disease (28)** | Inflammatory bowel disease  Crohn’s disease  Ulcerative colitis | 1461  1462  1463 |
| **Migraine (29)** | Migraine | 1265 |
| **Chronic sinusitis (30)** | Chronic sinusitis | 1416 |
| **Anorexia or bulimia (31)** | Anorexia, bulimia/other eating disorder | 1470 |
| **Bronchiectasis (32)** | Bronchiectasis | 1114 |
| **Parkinson's disease (33)** | Parkinson's disease | 1262 |
| **Multiple sclerosis (34)** | Multiple sclerosis | 1261 |
| **Viral Hepatitis (35)** | Infective/viral hepatitis  Hepatitis B  Hepatitis C  Hepatitis D  Hepatitis E | 1156  1579  1580  1581  1582 |
| **Chronic liver disease (36)** | Oesophageal varices  Non infective hepatitis  Liver failure/cirrhosis  Primary biliary cirrhosis | 1141  1157  1158  1506 |
| **Osteoporosis~ (37)** | Osteoporosis | 1309 |
| **Chronic fatigue syndrome~ (38)** | Chronic fatigue syndrome | 1482 |
| **Endometriosis~ (39)** | Endometriosis | 1402 |
| **Meniere disease~ (40)** | Meniere disease | 1421 |
| **Pernicious Anaemia~ (41)** | Pernicious anaemia | 1331 |
| **Polycystic ovaries~ (42)** | Polycystic ovaries | 1350 |
| **Cancer*** | Lifetime diagnosis |  |

* Cancer diagnoses were not included in the morbidity count for this study; resulting in a total of 42 morbidities included.

**Supplementary Table 3: Odds ratio (OR) of having malnutrition, probable sarcopenia, sarcopenia, (pre-)frailty by increasing multi-morbidity in all adults with cancer (N=4122)**

|  | **One long-term condition in addition to cancer diagnosis^1^** | | | **Two or more long-term conditions in addition to cancer diagnosis^1^** | | |
| --- | --- | --- | --- | --- | --- | --- |
| **Condition** | **OR** | **95% CI** | **p-value** | **OR** | **95% CI** | **p-value** |
| Malnutrition | 1.72 | (1.31, 2.30) | <0.0005 | 2.41 | (1.85, 3.14) | <0.0005 |
| Pre-frailty/frailty | 1.43 | (1.24, 1.68) | <0.0005 | 2.03 | (1.73, 2.38) | <0.0005 |
| Probable sarcopenia/sarcopenia | 1.21 | (0.88, 1.67) | 0.239 | 1.34 | (0.98, 1.83) | 0.066 |
| Probable sarcopenia alone | 1.21 | (0.88, 1.67) | 0.244 | 1.33 | (0.97, 1.84) | 0.080 |
| Sarcopenia alone | 1.36 | (0.53, 3.46) | 0.520 | 2.36 | (1.00, 5.54) | 0.049 |

**Supplementary Table 4:** **Odds ratio (OR) of having malnutrition, (probable-)sarcopenia, (pre-)frailty by increasing multi-morbidity in adults with a high cachexia risk cancer (N=689)**

|  | **One long-term condition in addition to cancer diagnosis^1^** | | | **Two or more long-term conditions in addition to cancer diagnosis^1^** | | |
| --- | --- | --- | --- | --- | --- | --- |
| **Condition** | **OR** | **95% CI** | **p-value** | **OR** | **95% CI** | **p-value** |
| Malnutrition | 2.91 | (1.65, 5.12) | <0.0001 | 2.85 | (1.62, 5.01) | <0.0001 |
| Pre-frailty/frailty | 1.84 | (1.25, 2.74) | 0.002 | 2.22 | (1.49, 3.31) | <0.0005 |
| Probable sarcopenia/sarcopenia | 1.93 | (0.92, 4.05) | 0.079 | 1.26 | (0.58, 2.75) | 0.552 |
